# Clinical Trials: Minimising source data queries to streamline endpoint adjudication in a large multi-national trial

**DOI:** 10.1186/1745-6215-12-112

**Published:** 2011-05-06

**Authors:** Elizabeth P Tolmie, Eleanor M Dinnett, Elizabeth S Ronald, Allan Gaw

**Affiliations:** 1 Glasgow Clinical Research Facility, Tennent Building, Western Infirmary, Glasgow, UK; 2 NHS Greater Glasgow & Clyde Pharmacovigilance Office, Robertson Centre for Biostatistics, Boyd Orr Building, Glasgow, UK; 3 Scottish Stroke Research Network, Walton Building, Glasgow Royal Infirmary, Glasgow, UK

## Abstract

**Background:**

The UK Clinical Trial Regulations and Good Clinical Practice guidelines specify that the study sponsor must ensure clinical trial data are accurately reported, recorded and verified to ensure patient safety and scientific integrity. The methods that are utilised to assess data quality and the results of any reviews undertaken are rarely reported in the literature. We have recently undertaken a quality review of trial data submitted to a Clinical Endpoint Committee for adjudication. The purpose of the review was to identify areas that could be improved for future clinical trials. The results are reported in this paper.

**Methods:**

Throughout the course of the study, all data queries were logged. Following study close out, queries were coded and categorised. A descriptive and comparative analysis was conducted to determine the frequency of occurrence for each category by country of origin.

**Results:**

From 1595 endpoint packages reviewed, 782 queries were generated. No source data queries were generated for countries with ≤ 25 recruited subjects, but both low recruiting and high recruiting countries had a high number of queries relating to subject identifiers.

**Conclusions:**

The implementation of some simple measures could help improve data quality and lead to significant savings.

## Background

The integrity of the results from clinical trials of investigational medicinal products (CTIMPs) is dependent on robust and credible data. Poor quality data may make it difficult for sponsors and research teams to demonstrate that they have met their legal and professional responsibilities in research. We have recently undertaken a quality review of the data submitted for adjudication to the Clinical Endpoint Committee (CEC) of a large international double blind, placebo controlled IIIb randomised trial. The study recruited 2,776 subjects from 280 centres in 25 countries [1].

The endpoint adjudication process was co-ordinated and supported by the Clinical Trials Unit (CTU), Glasgow Royal Infirmary, Glasgow, UK. The CEC, who were independent of the study sponsor and blinded to the treatment allocation, reviewed all causes of death, strokes and myocardial infarctions identified by the investigator as potential clinical endpoints; revascularisations were adjudicated by the study physician. Endpoints were classified according to definitions in the approved study protocol with the classification based on data from the Case Report Form and appropriate source documents (detailed in Clinical Endpoint Reporting Guidelines). Full details of the endpoint adjudication process are described elsewhere (submitted for publication).

To clarify the data requirements of the CEC and minimise the number of queries likely to occur, the CTU provided training to the sponsor study teams before trial start up and during the conduct of the trial. The data received from the sponsor was reviewed by the CTU team on an ongoing basis. Following trial close out, a full review was undertaken. This paper describes the purpose of the review, the methods used to conduct the review, and the results of the review. Based on the results, we offer some recommendations for actions that could reduce the cost associated with incomplete or inadequate data sets in a large international randomised controlled trial.

## Methods

### Data Tracking

**Figure 1:**
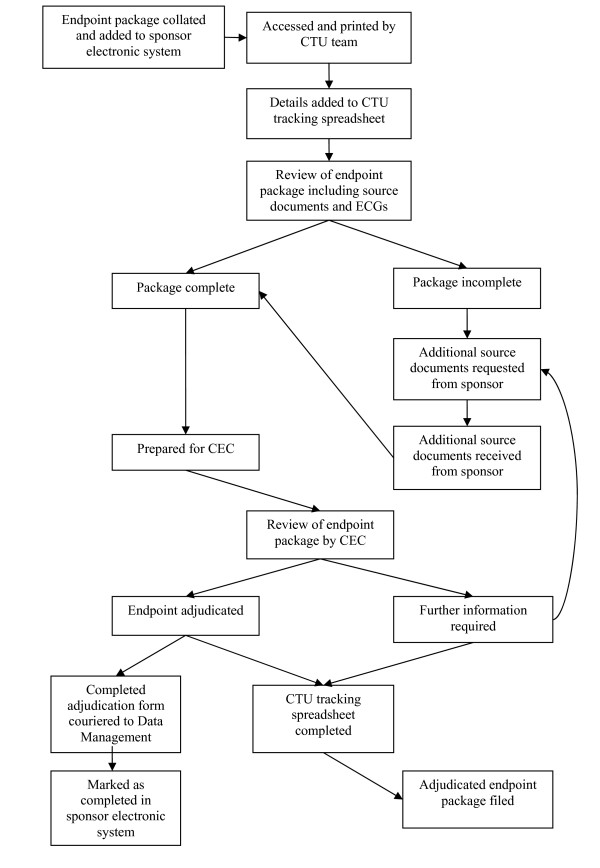
**Clinical Trials Unit Endpoint Review Process**.

In preparation for the trial start up, a system was devised for tracking all data received from, and sent to, the study sponsor endpoint office. CTU study specific Standard Operating Procedures specified the processes that were to be followed for the receipt, documentation, review, follow up and quality control of the data in order to ensure that each endpoint package was handled in accordance with Good Clinical Practice [2]. The process permitted all data related queries to be recorded, monitored, and followed up (Figure 1).

### Data Review

Following identification of a potential endpoint by an investigator, the sponsor endpoint office collated and sent an endpoint package containing the appropriate Case Report Form pages and a predetermined set of source documents to the CTU for adjudication. All documents received from the study sponsor were reviewed by the team at Glasgow to ensure they were complete, adequate, de-identified, legible, and consistent. Queries generated during the review were submitted to the sponsor as soon as identified. Those which were unanswered by the next working day were logged on to a database and the response date entered as appropriate. The database was collated monthly; outstanding queries were re-submitted to the sponsor at monthly or two monthly intervals. Where possible, data queries were resolved before the endpoint packages were submitted to the CEC for adjudication.

Following study close out, a review of all data queries generated by the CTU team over the course of the study was undertaken. The purpose of the close out review was to identify whether there were any recurring issues that impacted on the quality of the data received for adjudication, or on the adjudication decision. The primary objectives of the review were to:

• Identify data quality issues

• Determine whether the issues were country specific

• Determine whether any data queries delayed adjudication

• Identify processes that might improve data quality and the efficiency of the adjudication process in future clinical trials.

### Data Preparation and Analysis

All identified and logged data queries were entered on to the CTU study database in text format. These were then categorised by type and coded numerically. A descriptive analysis was conducted to identify the number of queries overall and the frequency with which each category occurred. A comparative analysis by country of origin was then conducted to determine whether there were any significant between-country differences. The results are discussed below.

## Results

### Number and Frequency of Queries Generated

Over the course of the study, the CEC reviewed 1595 endpoint packages. Packages could contain information for more than one endpoint event. From these, 782 data queries were generated; 164 (21%) of which were re-submitted to the sponsor on more than one occasion. Most packages generated only one query (n = 617); 165 generated more than one. The time between the query being submitted to the sponsor and being resolved ranged from one day to 22.8 weeks (Mean days = 51.9, SD 88.3; Median 23, IQR, 1.61).

### Categories of Queries Generated

For the purpose of identifying whether there were any consistent or recurring issues, queries were initially grouped into seven main categories as shown in Table 1. Figures for data that were received without being de-identified were not collected from the start of the trial and are therefore not included.

**Table 1:** Number and percentage of queries generated (all queries)

Type of query	Frequency	Relative %
Unique Subject Identifiers^1^	115	14.7

Missing baseline or event ECGs	117	15.0

Missing Troponin^2^/cardiac enzymes	83	10.6

Missing results/reports^3^	131	16.8

Illegible or obscured data	105	13.4

Dates/signatures^4^	181	23.1

Additional information^5^	50	6.4

Total	782	100

Erroneous or inconsistent^1^; level, type, or lower limit of detection^2^; neurological or cardiac imaging^3^; erroneous, missing, or inconsistent dates; missing signatures^4^; insufficient clinical information to permit adjudication^5^.

As illustrated, one half of the queries (51%, n = 401) related to inconsistent, erroneous or missing dates, missing signatures, illegible data or errors in unique subject identifiers (which enable data linkage while ensuring subject anonymity). Forty three percent (n = 331) related to missing source data, specified in the protocol and Clinical Endpoint Reporting Guidelines as necessary for adjudication. Some of the queries were generated in response to clinical statements in the source data that suggested a clinical event had occurred, or an investigation had been performed, but no associated result or report was included in the endpoint package. The remainder related to 50 requests from the CEC for additional clinical information after its initial review of the endpoint package. The former resulted in the adjudication decision being deferred on 39 occasions.

### Comparison of Type of Query by Country of Origin

Data were further combined to five categories to enable a statistical comparison to be made. An overall comparison of the number and type of data queries across participating countries suggested that at least one country was statistically significantly different from the others (P < 0.0001) (Kruskal-Wallis all pair-wise comparison, adjusted for ties). Subsequent pairwise comparisons revealed a number of between-country differences (P < 0.001). In most cases the wide confidence interval (CI) indicated a non significant result (CI crossed zero). Thus, there was no clear indication that one country differed from all others. The proportion of queries in each category differed within and across the participating countries (Table 2). For example, queries relating to unique subject identifiers were encountered most frequently in data from Italy and were the most problematic issue for Italy. Conversely, queries relating to dates or signatures were infrequent in Italy compared to most other countries including Iceland, a country with one of the lowest reported endpoint events.

**Table 2:** Type and proportion of queries generated from data received from participating countries

Type of query	Unique subject identifiers	Source Documents (Results/reports)	Clarification/legibility	Dates/Signatures	Additional clinical Information
**Country**	(Relative %)	(Relative %)	(Relative %)	(Relative %)	(Relative %)

Australia	20.5	48.7	15.4	10.3	5.1

Austria	10.8	56.7	10.8	21.6	0

Belgium	0	33.3	20.8	33.3	12.5

Canada	7.1	54.8	14.3	21.4	2.4

Czech Rebublic	10.7	50.0	17.9	14.3	7.1

Denmark	9.7	35.5	6.4	41.9	6.4

Finland	22.2	51.8	14.8	7.4	3.7

France	10.3	48.3	13.8	17.2	10.3

Germany	14.0	35.1	10.5	31.6	8.8

Hungary	12.3	56.1	10.5	19.3	1.7

Iceland	0	0	0	100	0

Netherlands	10.5	38.6	15.8	24.6	10.5

Norway	11.1	44.4	11.1	22.2	11.1

Poland	11.4	50.0	11.4	16.0	11.4

Sweden	15.4	53.8	15.4	11.5	3.8

Switzerland	16.7	83.3	0	0	0

UK/Ireland	17.5	37.5	6.2	30.0	8.7

Brazil	15.3	25.0	23.6	29.2	6.9

Bulgaria	33.3	29.2	12.5	25.0	0

Greece	0	100	0	0	0

Italy	40.0	33.3	13.3	6.7	6.7

Korea	30.8	30.8	7.7	30.7	0

Mexico	25.0	16.7	25.0	29.2	4.2

Turkey	0	33.3	0	66.7	0

**Total**	14.4	43.6	11.6	25.4	5.1

The exception to this was Switzerland which had no date or signature queries but the highest proportion of source document queries (83%). This was followed by Greece, Austria, Hungary, Canada and Finland where more than 50% of the queries related to source data.

### Reasons for Delayed Response to Queries

A full review of the data was conducted to determine whether there was a consistent explanation for delays of more than 90 days. The reason for the delay was not reported in many cases. Where the data were available, the delay was identified as being due to events that had not been anticipated at the study planning stage. These included:

• Difficulty in accessing source data relating to a clinical event where the trial subject was admitted to a non-participating hospital.

• The requirement for the CEC to have access to both original language and translated documents from each participating country.

• Identification of clinical events by the CEC or CTU reviewer that had not been identified or reported by the sponsor study team.

• Delay in query response time due to transfer of study management responsibility (and outstanding queries) from one team and/or country to another while the study was in progress.

## Discussion

ICH GCP Guidelines [2] specify that clinical trial data must be accurately reported, recorded, and verified to ensure its scientific integrity. Our review revealed a number of data errors and inconsistencies that occurred repeatedly, despite being highlighted to the sponsor at an early stage and at intervals during the study. Although the delay in the resolution of queries had no impact on the trial results, it did have an impact on timelines and was resource intensive for the sponsor as well as the CTU team. Some of the delays were due to issues that had not been anticipated at the study planning stage and, hence, could not have been avoided. They do, however, highlight the need to give some consideration to their potential impact when planning future studies.

It is possible that some of the delays incurred were due to sub optimal staffing levels, high staff turnover, particularly as the study neared completion, or different work practices (such as vacation times) in some countries. It is also possible that some of the between country differences were due to the national characteristics of individual health care systems. In some cases, the geographical spread of study sites or low resource investment may have been problematic for monitors when arranging site visits to check source data or obtain required information. The qualifications or professional background of the monitor may also be influential. Some countries employ clinically qualified staff in monitoring roles whereas others do not; this may have a positive or negative impact on the data collected.

A well planned prospective study would be required to identify any statistically significant and more subtle differences between sites/and or countries and the cost implications of addressing these.

A small number of data errors at one site may not have a negative impact on the conduct of a trial or data integrity. However, when the problem occurs over a number of sites the cumulative effect may be great and the financial impact extensive. It is imperative that these issues are addressed.

It is important to note that the data quality issues discussed in this report were identified at a late stage in the data collection and review process, that is, when the endpoint packages were submitted for adjudication. At that point, the data had been exposed to a series of monitoring procedures and data checks. As the results do not include figures for data queries that were resolved quickly (within one working day), or data that was not de-identified before being sent for adjudication, the extent of the problem is underestimated. Moreover, transferring identifiable patient data from clinical sites to the sponsor or clinical research organisations breaches the Principles of Good Clinical Practice [2] and the data protection legislation in the UK [3] and elsewhere. These are issues that clearly need to be addressed. Based on the results of our analysis we, therefore, make the following recommendations:

• In addition to Good Clinical Practice training, all members of the clinical research team should be trained in the requirements of the trial before study start up. This would ensure that all staff are aware of their trial specific responsibilities and data reporting requirements.

• New members of the research team should be trained in the content of the study protocol and study specific documentation requirements of the trial before they are required to collect, handle, or review data. This would minimise the risk to data quality, particularly where there may be a high turnover of research support staff.

• Providing those who are new to research with a mentor until they are competent to fulfil their role is likely to be an effective way to reduce cost and drive up the quality of the data.

• Study specific endpoint data and documentation requirements should be accessible to trial staff at all times. This would provide a reference point for any queries that might arise and thus circumvent potential errors.

• A system should be put in place which permits changes to study requirements to be disseminated to all trial staff in a timely and effective manner. This would ensure that all trial staff are kept informed of current documentation processes and procedures. A system that alerts trial staff to any required amendments, or protocol changes, and associated data requirements, and acknowledges receipt of these, would be advantageous.

• Where possible, an electronic rather than paper based CRF should be used. This would reduce the potential for errors by preventing the submission of incomplete data, or data that exceeds pre-determined parameters, and reduce the need for double entry.

• When the source documents required to support a study endpoint are being agreed, consideration should be given to how accessible the required documents will be to trial staff.

• A simple data tracking system established at an early stage in the trial set up would identify any recurrent queries and allow these to be addressed.

## Conclusions

The results of this analysis highlight the need to implement measures that will reduce the problems associated with the collection of data required to support the endpoint adjudication process in large multicentre trials. Adequate and appropriate training of all staff involved in the process before study start up and throughout its duration will eliminate many of the minor problems identified. A simple data tracking system established at an early stage in the trial set up would identify any recurrent queries and allow these to be addressed. As data sent for review and adjudication without being adequately de-identified were not logged from the date of study start up, the results may underestimate the full extent of the problem.

Whether our recommendations will save time or money is likely to depend on the nature of the study, what is implemented and how it is implemented. The resources spent on the implementation of any of these recommendations would need to be justified. A complex trial may require more training resources. However, ensuring that submitted documents contain all the required signatures and dates could resolve some of the issues relatively easily and cost effectively. For all studies, any reduction in the time and effort spent retrieving missing or incomplete clinical trial data should result in cost savings.

## List of Abbreviations

CEC: Clinical Endpoint Committee; CI: Confidence Interval; CTU: Clinical Trials Unit; GCP: Good Clinical Practice; ICH: International Conference on Harmonisation; SD: Standard Deviation

## Competing interests

The authors declare that they have no competing interests.

## Authors' contributions

EPT and EMD conceived the review and carried out the analysis, EPT and EMD drafted the manuscript. ESR and AG reviewed the manuscript. All authors read and approved the final manuscript.
